# Molecular Identification and Acid Stress Response of an *Acidithiobacillus thiooxidans* Strain Isolated from Rio Tinto (Spain)

**DOI:** 10.3390/ijms241713391

**Published:** 2023-08-29

**Authors:** Ana Ibáñez, Carlos Barreiro, Alba Diez-Galán, Rebeca Cobos, Carla Calvo-Peña, Juan José R. Coque

**Affiliations:** 1 Instituto de Investigación de la Viña y el Vino, Escuela de Ingeniería Agraria, Universidad de León, 24009 León, Spain; aibas@unileon.es (A.I.); aldig@unileon.es (A.D.-G.); rebeca.cobos@unileon.es (R.C.); ccalp@unileon.es (C.C.-P.); 2 Instituto Tecnológico Agrario de Castilla y León (ITACYL), 47071 Valladolid, Spain; 3 Área de Bioquímica y Biología Molecular, Departamento de Biología Molecular, Universidad de León, 24071 León, Spain

**Keywords:** *Acidithiobacillus thiooxidans*, 2D-DIGE, RT-PCR, omics, pH stress, mass spectrometry

## Abstract

*Acidithiobacillus thiooxidans* is of paramount importance in the development of biomining technologies. Being widely recognized as an extreme acidophile, extensive research has been dedicated to understanding its significant role in the extraction of several ores in recent years. However, there still exist significant molecular uncertainties surrounding this species. This study focuses on developing a taxonomic assignment method based on the sequencing of the *16S-5S rRNA* cluster, along with a qPCR-based technology enabling precise growth determination. Additionally, an approach to understanding its response to acid stress is explored through RT-PCR and MALDI-TOF analysis. Our findings indicate that when subjected to pH levels below 1, the cell inhibits central (carbon fixation and metabolism) and energy (sulfur metabolism) metabolism, as well as chaperone synthesis, suggesting a potential cellular collapse. Nevertheless, the secretion of ammonia is enhanced to raise the environmental pH, while fatty acid synthesis is upregulated to reinforce the cell membrane.

## 1. Introduction

The mining sector plays a crucial role in the global economy. Many industries depend on the supply of mineral raw materials, such as iron (construction), aluminum (automotive), or potash (agriculture). In fact, the data provider company Statista (https://es.statista.com, accessed on 1 June 2023) states that the revenue of the top 40 global mining companies amounted to approximately USD 943 billion in 2022. However, significant losses of valuable minerals occur due to inefficient processes and the inability to extract from low-grade waste zones, amounting to billions annually [1,2,3,4,5]. Consequently, hundreds of tons of minerals are deposited annually as wastes in dumps; such is the case with phosphorus mining, where bulk sediments contain a mass fraction of P_2_O_5_ ranging from 4 to 21% of the total weight [6], or in the copper industry, where more than 8 g of Cu per kg of residue are lost [7].

In addition to the imperative need for high-quality raw materials, conventional mining has numerous well-known environmental disadvantages, including soil and water contamination by heavy metals from acid mine drainage, large carbon footprints, pollutant emissions, and erosion [8]. As a result, the concept of sustainable mining has gained attention since the early 1990s [3], focused on the new concept of biomining. It is an emerging eco-friendly technology that utilizes biological systems, specifically microorganisms, to facilitate the extraction and recovery of metals and other substances with industrial interest from low-grade ores and ores that contain elevated concentrations of contaminants [9]. It is perceived generally as a green process, since the microorganisms involved in mineral oxidation processes are typically autotrophs fixing CO_2_, in contrast to traditional mining and smelting operations that emit significant amounts of carbon dioxide [10,11,12]. The most frequently used are the so-called sulfur-oxidizing bacteria (SOB), which convert sulfur (S) to sulfuric acid, thereby acidifying soils and mobilizing metal ions. Numerous SOB strains are becoming relevant in biomining and bioremediation industries, such is the case of *Acidithiobacillus ferrooxidans*, *Acidithiobacillus thiooxidans*, *Thiobacillus thioparus*, and *Sulfobacillus thermosulfidooxidans.* However, in some cases, when minerals are not effectively solubilized by protons alone, the activity of SOBs may be insufficient. In such cases, iron-oxidizing microorganisms such as A. ferrooxidans assume a pivotal role in biomining processes [13,14,15]. Biomining microorganisms are widely distributed in acidic sulfur-containing environments in soil and water, actively participating in the global cycles of sulfur and iron [8,16,17]. *Acidithiobacillus* (formerly *Thiobacillus*) is the most relevant genus [18], which mainly comprises autotrophic and moderately or extremely acidophilic species that utilize sulfur or iron as an electron donor, with the consequent production of sulfuric acid [19]. However, despite their relevance in both natural and industrial processes, most of the molecular mechanisms involved in survival at superacid pH levels remain uncertain as a result of handling problems.

*A. ferrooxidans* originally emerged as the acidophile model microorganism due to its undeniable applications in copper biomining [20], and several multi-omic approaches have been carried out in recent years to study its genetic makeup [21,22,23,24]. However, the strain *A. thiooxidans* has recently attracted research interest, albeit with relatively slow progress. Nowadays, several studies describe this strain as a notorious workhorse for the industrial extraction of Cu, Ni, Zn, Co, and Au from different ores and residues [25,26,27]. However, the members of this genus exhibit certain limitations that hinder their industrial application, including the following: (i) long growth cycle, (ii) slow bioleaching rate, and (iii) harsh conditions for growing under controlled conditions. Many studies propose genetic improvement strategies to address these drawbacks, although it is quite a challenge, due to the limited molecular knowledge [27]. Furthermore, the identification and classification of the *Acidithiobacillus* strains is a jigsaw puzzle to date, because genetic markers are still too scarce to allow a precise identification of the isolates. Additionally, it is considered that some of the *Acidithiobacillus* strains in the existing databases may be misclassified or unassigned, warranting further investigation [28,29,30,31,32]. Initially, they were considered as members of the β-proteobacteria class. Later, the *Acidithiobacillus* genus was reassigned to ƴ-proteobacteria. Finally, it was positioned as a sister class to the β-, ƴ-, and ƹ-proteobacteria, along with *Thermithiobacillus tepidarius*, forming the new class Acidithiobacillia. Therefore, there is a considerable gap in the knowledge of these fascinating extremophile microorganisms [31].

This study provides comprehensive insights into the growth of *Acidithiobacillus* strains under upgraded cultured conditions. It focuses on their taxonomical classification and reveals the genetic mechanisms that enable their survival in highly acidic environments, such as those found in biomining and bioleaching systems. By improving our understanding of their accurate identification and optimal growth conditions, this information holds great potential for enhancing their industrial applications.

## 2. Results

### 2.1. Isolation and Identification of A. thiooxidans Lo.19II-12

*A. thiooxidans* Lo.19II-12 was isolated from sludge samples from the Rio Tinto enriched in *Acidithiobacillus thiooxidans* medium for 1 week to facilitate its isolation. The isolation process was conducted on *Thiobacillus* agar supplemented with bromocresol green to detect the production of sulfuric acid by the isolates. However, most chemolithoautotrophic organisms are highly sensitive to the presence of organic compounds in culture media, which makes the isolation of sulfur-oxidizing bacteria (SOB) strains a complex process. Agar is a polysaccharide that hydrolyses under acidic conditions, and the galactose produced from its hydrolysis inhibited the growth of SOB during the initial isolations [33,34].

On the contrary, a total of eight morphologically distinct sulfur-oxidizing bacteria were obtained when agarose was used as a gelling agent for solid media. All eight strains were selected based on their ability to produce sulfuric acid in the medium, and they were further subjected to identification. 

The identification of all isolates was based on their *16S rRNA* gene sequencing. However, the results from the BLASTn software (https://blast.ncbi.nlm.nih.gov/Blast.cgi?PROGRAM=blastn&BLAST_SPEC=GeoBlast&PAGE_TYPE=BlastSearch, accessed on 14 August 2023) at were inconclusive for all isolates, providing a genus-level identification, but not a species determination. Three strains were identified as *Thiomonas* sp., and the rest of the isolates, including Lo.19II-12, were identified as Acidithiobacillus members (Table S1). The intra- and inter-specific variation along the 16S rRNA gene within the *Acidithiobacillus* genus highlights the need for new markers to achieve accurate taxonomy classification [35,36].

Due to this rationale, the entire genome of *A. thiooxidans* Lo.19II-12 was subjected to sequencing. Nonetheless, the potential was also considered to identify a novel marker that could enable differentiation among species within the *Acidithiobacillus* genus, avoiding complete genome sequencing in future studies.

### 2.2. 16S-5S Ribosomal Cluster Alignment Analysis

The genus *Acidithiobacillus* has been traditionally classified into different clades that group one or several species. Nuñez et al. [37] performed such classification using four clades, although their analysis did not include isolates of *A. sulfuriphilus* and *A. ferrianus*. Subsequently, Moya-Beltrán [31] classified the genus into two clades (one for *A. caldus* and another for the rest of the species). However, the analysis included diverse genera in addition to *Acidithiobacillus*, which may influence isolate clusterization.

Traditionally, the *16S rDNA* sequencing results for the *Acidithiobacillus* genus have been inconclusive, with only 75% of the isolates being assigned to the species level [37]. Different strategies have been proposed previously, such as Multilocus Sequence Analysis (MLSA) [36] or the 16S oligotyping [37]. In this work, the alignment of the *16S-5S rRNA* cluster was the selected methodology.

**Figure 1:**
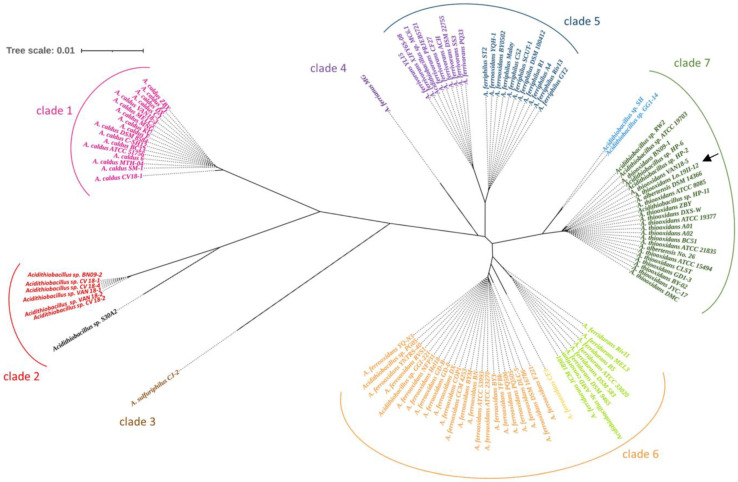
Phylogenetic tree based on *16S-5S rRNA* cluster of the isolate Lo.19II-12 relative to 104 *Acidithiobacillus* strain sequences from GenBank database. Species affiliation are as follows: *A. caldus* (pink), *A. thiooxidans* (dark green), *A. ferrianus* (dark purple), *A. ferrivorans* (purple), *A. ferriphilus* (dark blue), *A. ferridurans* (light green), *A. ferrooxidans* (orange), and *A. sulfuriphilus* (brown). The tree was inferred using the neighbor-joining method [40]. The optimal tree with the sum of branch length = 0.08465532 is shown. The tree is drawn to scale and the evolutionary distances were computed using the maximum composite likelihood method [41]. Evolutionary analyses were conducted in MEGA7 [42], and the tree was visualized using iTOL [43]. *A. thiooxidans* Lo.19II-12 is highlighted with a black arrow.

The taxonomic analysis of the *16S-5S rRNA* cluster from the 104 *Acidithiobacillus* strains plus the isolate Lo.19II-12 revealed their grouping into seven different clades (Figure 1). The first clade consists of *Acidithiobacillus caldus* strains, whereas clade two encompasses a group of unclassified species, including BN09-2, CV18-2, CV18-3, VAN18-1, and VAN18-2. The third clade exclusively contains the *Acidithiobacillus sulfuriphilus* CJ2 strain, which remains the only described strain of its species to date [38]. Clade four includes strains of *Acidithiobacillus ferrianus*, with *A. ferrianus* MG being the sole representative recently described by Norris et al. [39]. Clade five clusters both *Acidithiobacillus ferrivorans* and *Acidithiobacillus ferriphilus* strains, while clade six encompasses *A. ferrooxidans* and *Acidithiobacillus ferridurans*. Last, clade seven includes members from *A. thiooxidans* and *Acidithiocabillus albertensis* species.

Based on this phylogenetic tree, several unclassified strains have been successfully classified into specific clades, alongside previously described species, as well as some strains have been re-classified under a different species. This finding suggests that the taxonomic classification of sequences deposited in databases needs to be carefully revisited and re-evaluated:

- Clade five compiled two different groups: (i) *A. ferrivorans* strains (including all *A. ferrivorans* strains and the unclassified strain MC6.1) and (ii) *A. ferriphilus* strains (comprising all *A. ferriphilus* strains, along with the strains of *A. ferrooxidans* BY0502 and YQH 1).
- Clade six comprises the remaining strains of *A. ferrooxidans*, along with the unclassified strains PG05 and GGI 221, the strains of *A. ferridurans*, and the unclassified strain AMD consortium.
- Clade seven joined two groups: (i) the strains belonging to both *A. thiooxidans* and *A. albertensis* species, along with some unclassified strains such as ATCC 19,703, HP-2, HP-6, HP-11, and RW2, and (ii) two unclassified strains: GG1-14 and SH.

The taxonomic classification based on the *16S-5S rRNA* cluster unequivocally places Lo.19II-12 isolate within clade seven, identifying it as *A. thiooxidans*. In contrast to *16S rRNA* sequencing, this classification offers strong support for the precise taxonomic assignment of the isolate.

### 2.3. Determination of A. thiooxidans Lo.19II-12 Growth by qPCR

Once the isolate was taxonomically classified, one of the main challenges when working with *Acidithiobacillus* strains is to achieve an accurate determination of its growth. Based on previous analyses, the determination of *A. thiooxidans* Lo.19II-12 growth through traditional methods such as optical density (DO_600nm_), cfu (colony-forming unit) determination on agar plates, or hemocytometer count was discarded due to the inconsistency in the final results. A previously developed method for *Streptomyces* [44] was successfully adapted to *Acidithiobacillus* strains. Thus, the proposed protocol enables the determination of the cell count in a sample based on the C_t_ value obtained from the q-PCR amplification of the target sequence, as well as the length and the copy number of the target gene.

**Figure 2:**
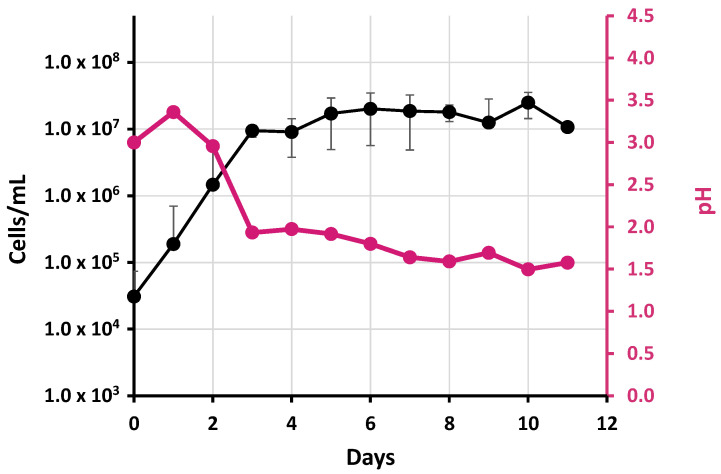
Growth estimation of *A. thiooxidans* Lo.19II-12 strain by measuring number of cells in suspension in liquid cultures developed in DSMZ 35 medium at 30 °C and 200 rpm for 11 days as determined by qPCR. The pH of the supernatant is shown on the secondary axis. Data (with SD) are the average of three independent experiments.

After the transformation of C_t_ values to cell concentration (see material and methods), *A. thiooxidans* Lo.19II-12 showed an exponential phase of 3–4 days, reaching a cell concentration close to 10^8^ cells/mL (Figure 2). Interestingly, the observed growth was higher and faster than what has been previously reported by other authors (see discussion below). Throughout this period, there was a significant drop in the pH of the culture medium, which decreased from day 1 to day 3 when the culture entered the stationary phase. The pH continued to slowly decline until the end of the incubation, reaching a final pH of around 1.5. 

### 2.4. Determination of the pH-Stress Conditions by RT PCR

To analyze the pH stress in *A. thiooxidans* Lo.19II-12, several genes involved in stress response were used as reporters. Thus, three well-known chaperones (*groEL2*, *dnaJ*, and *dnaK*) from the so-called heat shock proteins (Hsps), whose function is to protect proteins against protein aggregation, were studied. Additionally, *dna* genes (such as *dnaJ* or *dnaK*), which are related to the adaptive cellular response in many stress conditions (including acid stress in *Acidithiobacillus* [45,46]), were also a target of the analysis. Finally, the *rpoH* gene, which encodes the σ32 factor involved in the regulation of the expression of heat shock and other stress genes [47], was also selected. pH 0.5 and 1.5 were selected as “acidic conditions”, whereas pH 3.0 was the control condition and pH 5.5 and 6.0 were the “basic conditions”.

Contrary to previous findings, none of the selected genes showed up-regulation under acid stress conditions (pH 0.5 or 1.5) after 30 or 60 min of stress (Table 1) [45]. Interestingly, *A. thiooxidans* Lo.19II-12 exhibited a decrease in the expression of these genes after 30 min at pH 0.5 and a complete cessation of their expression after 60 min. When incubated at pH 1.5, the cells initially reduced the expression of *dnaJ* during the first 30 min, but the expression was reactivated after 60 min. However, the opposite trend was observed for *rpoH*, which exhibited overexpression during the initial 30 min, followed by a cessation of this overexpression after the 60 min period.

In contrast, when subjected to moderate pH conditions (pH 6.0), *A. thiooxidans* Lo.19II-12 responded by activating the expression of *dnaJ* and, particularly, the *rpoH* gene after 60 min. In the case of pH 5.5, the initial response involved a reduction in the expression of the genes *dnaJ*, *rpoH*, and *dnaK*, although the expression levels for *dnaJ* and *rpoH* subsequently returned to normal levels after 60 min of incubation.

This suggests that, despite its typical pH range of 0.5–5.5 [19], *A. thiooxidans* Lo.19II-12 is capable of surviving at pH 6.0, possibly by overexpressing different survival mechanisms that involve the chaperone genes. However, it seems unable to survive at pH 0.5, as evidenced by growth inhibition observed in these analyses. This phenomenon could be attributed to the destabilization of the capsule structure, as described by Feng and co-authors [45].

### 2.5. Proteome Analysis of A. thiooxidans Lo.19II-12 under pH Stress by 2D-DIGE Analysis

pH is a crucial parameter in biomining systems as it exerts a significant influence on *Acidithiobacillus* growth and oxidative capacity, subsequently impacting its leaching efficiency. Previous studies have reported the response of *Acidithiobacillus* strains to pH stress on cytoplasmic membrane composition [45,48,49]. However, the proteomic and transcriptomic analysis of SOB strains, especially *A. thiooxidans*, is a recent area of interest [46,50,51]. Based on the results observed in the RT-PCR, preliminary tests were carried out at pH 0.5, 0.7, 1.0, and 1.5 to determine the optimal conditions for the study of acid stress. Finally, pH 0.7 was determined as the extreme acid condition. Again, pH 3.0 was selected as the control, whereas pH 6.0 was chosen as the “basic” pH condition.

A total growth inhibition of *A. thiooxidans* Lo.19II-12 and loss of mobility were detected at pH 0.5 by observation under an optical microscope. These observations, which are in concordance with those of Feng et al. (2015), indicated that *A. thiooxidans* could collapse at pH 0.5. This fact results in an insufficient amount of protein to carry out the proteome assays. As a result, pH 0.7 was determined as the optimal condition for studying extreme acidic pH.

2D-DIGE analysis revealed 573 spots as differentially expressed (>2 or <−2 difference of expression ratio, and *p*-value < 0.05) when pH 0.7 vs. 3.0 were compared. However, no significant differences between pH 3.0 and 6.0 were observed. Once again, these findings suggest that *A. thiooxidans* Lo.19II-12 prefers a more neutral pH (6.0) rather than an extremely acidic pH such as 0.5. Thus, 201 spots were selected from the gel and 53 were identified, showing 38 different proteins. It was observed that central energetic metabolism was severely restricted in *A. thiooxidans* Lo.19II-12 under extremely acidic pH conditions (Table S2). The main routes affected were the following: (i) carbon metabolism and CO_2_ fixation, (ii) energy metabolism, (iii) nitrogen fixation, and (iv) stress response (chaperones).

**Figure 3:**
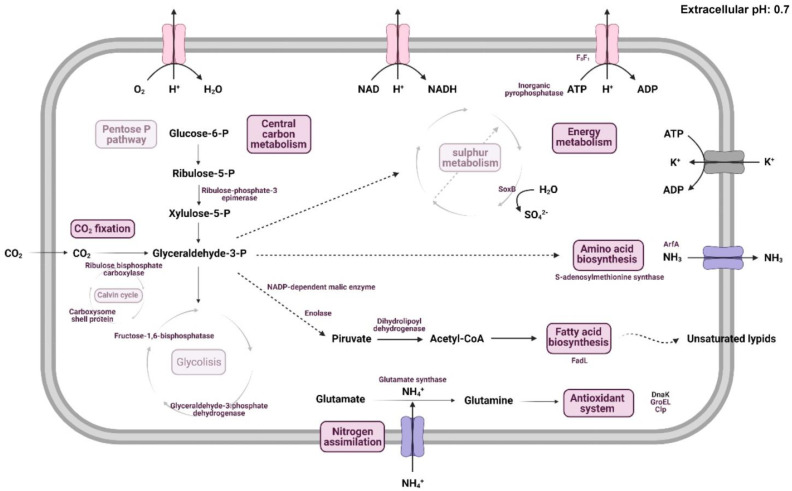
Model of *A. thiooxidans* metabolism and enzymes down- or up-regulated at pH 0.7. The enzymes that were identified by proteomic analysis are highlighted in purple in their respective metabolic pathways. The figure illustrates how *A. thiooxidans* adapts to acidic environments by regulating the activity of key enzymes involved in carbon metabolism and CO_2_ fixation, energy metabolism, nitrogen fixation, and stress response (chaperones).

CO_2_ fixation is closely related to the central carbon metabolism in chemoautotrophic bacteria as they utilize it as a carbon source for cell growth. Consequently, key enzymes involved in carbon fixation, such as carboxysome shell protein and ribulose-1,5-bisphosphate carboxylase (Rubisco), were observed to be down-regulated. Additionally, enzymes from the pentose phosphate pathway and glycolysis, including enolase, fructose-1,6-bisphosphatase, and ribulose-phosphate-3-epimerase, showed reduced expression levels (Figure 3).

The down-regulation of the energy metabolism was also evident under extreme acid conditions. Thus, the sulfur oxidation protein SoxB (from the *sox* gene family) was detected during the analysis. This enzyme is a thiosulfohydrolase which plays a crucial role in the oxidation of thiosulfate, and it is associated with sulfur metabolism in chemolithotrophs [52]. Regarding nitrogen fixation, the enzyme glutamate synthase, involved in NH_4_^+^ fixation into glutamate to produce glutamine, and associated with the antioxidant system, was also found to be down-regulated. Similarly, some of the identified chaperones, such as Clp or GroEL2, exhibited down-regulation at pH 0.7, chaperones which play a role in protein folding during the adaptive cellular response to various stress conditions, including extreme acid stress.

Similarly, the sliding clamp protein was also down-regulated. It is a ring-shaped protein that encircles duplex DNA, binds to the DNA polymerase, and tethers it to the DNA template, preventing its dissociation and providing high processivity. It plays an essential role in DNA repair, being crucial for cell viability, especially under stress conditions, when they also play a role in the repair of damaged DNA [53]. Finally, the L7/L12 ribosomal protein (located in the 50S ribosomal subunit) was also down-regulated, which could mean that the protein biosynthesis levels should be lower under extreme conditions. The same result was also reported for *A. ferrooxidans* under osmotic stress [54].

On the other hand, two typical mechanisms related to survival in extreme acid environments were detected as up-regulated: (i) the increase of the unsaturated acid composition of the outer membrane (due to the overexpression of FadL), and (ii) ammonia secretion (due to the overexpression of ArfA). On the one hand, FadL is a long-chain fatty acid transporter which has been previously reported in changes in cell membrane fatty acids in *A. thiooxidans* (and other *Acidithiobacillus* strains) during extreme acid conditions, with an increase in the unsaturated acid composition, especially the cyclopropane (C19-cyc) [48,51,55]. On the other hand, ammonia biosynthesis has also been identified as a target of pH stress by Yin et al. [51] in a transcriptomic analysis incubating *A. thiooxidans* under pH 0.8. Thus, L-glutamine is transformed to L-glutamic acid, releasing a molecule of ammonia during the process. In this case, the identified protein was ArfA, a peptidoglycan-binding protein which has been related to ammonia secretion in other strains to neutralize the pH of the medium in acid environments [56,57,58].

## 3. Discussion

The phylogenetic analysis of an *A. thiooxidans* isolate (Lo.19II-12) revealed misclassifications within the *Acidithiobacillus* genus. Traditionally, the genus *Acidithiobacillus* has been classified into four clades, including seven different species: *A. thiooxidans*, *A. ferrooxidans*, *A. caldus*, *A. albertensis*, *A. ferriphilus*, *A. ferrivorans*, and *A. ferridurans* [37], although two new species have been described recently: *A. sulfuriphilus* [38] and *A. ferrianus* [39]. However, multiple discrepancies have emerged in recent years regarding the current classification of the genus *Acidithiobacillus* [29,30,31,32,37,59]. Thus, to date, the taxonomic assignment of the *Acidithiobacillus* species has relied on classifications based on morphological and physiological characteristics, which have led to unassigned or misclassified strains for years [37]. In this study, the data led us to assist this hypothesis.

Nevertheless, all the phylogenetic analyses conducted thus far have been based on the sequencing of complete genomes, which entails complexity and substantial economic expenses. In contrast, our results indicate that while species-level identification using universal primers 27F and 1492R [60] may not be achievable [32,36,37], extensive genome sequencing is not essential. The ribosomal cluster 16S-5S has proven to be adequate for accurate classification.

Intraspecific divergence in *Acidithiobacillus* DNA sequences has been previously attributed to adaptations to specific ecological niches [61,62]. Nevertheless, recent MLSA and pan-genomic studies highlighted the need for reclassifying species within the genus [29,31]. The rapid evolutionary divergence of *A. caldus* has resulted in significant phylogenetic differences from other members of the genus [29,59]. Some authors even advocate for its exclusion from the genus and reclassification as *Fervidacidithiobacillus caldus* [31]. Besides, there is a need to define new species, as some strains do not correspond to any of the currently defined species. Such is the case for the unclassified strains CV18-1 and CV18-4 (clade two) or *A. sulfuriphilus* CJ-2 (clade three), which should be reclassified as *Igneacidithiobacillus copahuensis* and *Ambacidithiobacillus sulfuriphilus*, respectively, two newly species defined by Moya-Beltrán et al. (2021). Greater discrepancies exist around clade seven, where two genetically indistinguishable strains are grouped: *A. thiooxidans* and *A. albertensis*. In this case, while Moya-Beltrán [31] classifies *A. albertensis* DSM 14366 as *A. thiooxidans* sp. *albertensis* DSM 14366, Li et al. (2019) renamed it as *A. thiooxidans* DSM 14366. In any case, in accordance with previous results, the analysis of genome similarity has shown that there is insufficient divergence between both species to warrant their classification as different species. In conclusion, these results corroborate previous studies and provide an additional classification for strains that have not been analyzed previously (e.g., HP-2, HP-6, HP-11, and RW2), as well as highlight the need for a taxonomic reclassification of the genus (Table S3).

The accurate definition of *A. thiooxidans* growth is a key challenge traditionally tackled but not properly solved. In 1995, Konishi et al. [63] described the growth of *A. thiooxidans* for the first time, demonstrating an alternating pattern of growth in suspension and on the surface of sulfur particles present in the medium. Since that initial study, several manuscripts have reported the measurement of *Acidithiobacillus* growth by different methods, resulting in significant variations in the obtained results: (i) optical density, with values ranging from 0.05 to 0.1 [63,64,65], (ii) hemocytometer [66,67], (iii) cell counter cytometer [68], or even (iv) counting colony-forming units (cfu) on agar plates. However, *A. thiooxidans* growth on solid media poses challenges owing to its slow spread, autotrophic metabolism, and sulfuric acid production [27], which can impact the solubility of agar. Additionally, the optical density values were notably low, resulting in considerable deviations in the measurements. Similarly, the measurements using a hemocytometer also exhibited significant discrepancies between triplicates. Consequently, achieving precise quantification of growth proved unfeasible due to the pronounced variability in these values.

The adaptation of a previously described method for the Actinobacteria *Streptomyces* has detected a faster growth of *A. thiooxidans* in comparison to that determined by spectrophotometry. Consequently, the stationary phase is reached within 3 to 4 days, in contrast to the 12 to 14 days previously reported [63,65]. Similar results have been reported by Esparza et al. [69] for *A. ferrooxidans* growth and by Camacho et al. [68] for *A. thiooxidans*, with both reaching around 10^8^ cells/mL after 3 days of incubation, with significant variations observed between replicates (likely due to the presence of insoluble sulfur in the medium). This indicates that it is not a recommended method for accurately determining *Acidithiobacillus* growth [70]. Instead, qPCR has emerged as a well-known, reproducible, and sensitive technique for accurate quantification of *Acidithiobacillus* growth.

The main use of *Acidithiobacillus* strains is the development of biomining and bioremediation technologies. However, the evolution of these strains during the process is often overlooked. Biomining technologies typically operate under extremely acidic conditions with pH levels between 1.5 and 2.5 [71,72], which can impact *Acidithobacillus* growth and leaching rates [48,72]. Some *Acidithiobacillus* species, such as *A. caldus*, have shown adaptive capacity to these extreme acidic conditions and can grow at pH levels close to 1.5 [46], while *A. ferrooxidans* has been found to thrive at pH levels around 1.8 [22]. However, certain bioleaching operations (e.g., Monywa, Myanmar) operate at pH levels below 1.2, which can cause the collapse of microbial populations. Theoretically, a lower pH enhances the dissolution of minerals present in the rock. As a result, the concept of utilizing synthetic biology tools to design and construct more resilient biomining strains is gaining traction [9]. The primary focus of these studies is the improvement of resistance against acid stress. However, from a realistic standpoint, it is essential to bear in mind that the use of genetically modified strains is limited to enclosed environments, making them a viable solution in the development of biomining technologies within bioreactors. Nevertheless, the inability to release modified microorganisms sets different objectives when working on bioleaching dumps. It has been previously reported that the efficiency of the process is highly dependent on the ambient pH, which requires meticulous pH control to maintain it at optimal levels for bacterial growth and survival [73,74]. One promising approach to enhance the extreme acid tolerance involves employing adaptive laboratory evolution. Indeed, conducting serial passages of the strain under increased acidic conditions can potentially lead to the development of extremely acid-resistant strains. For example, *Metallosphaera sedula*, an acidophilic lithoautotrophic archaeon, demonstrated enhanced copper leaching activity after undergoing adaptive laboratory evolution to improve its acid resistance. In the same vein, the extremely thermoacidophilic *Sulfolobus solfataricus* demonstrated the ability to grow at pH 0.8 after undergoing a serial culture in which culture acidity was gradually increased.

Thus, contrary to theoretical expectations, the lowest pH does not always yield the best performance currently [73,75]. The dynamic ecosystem within the bioleaching dump sets the optimal conditions for achieving the highest efficiency. As stated earlier, pH stress represents the main challenge in bioleaching systems, and a comprehensive understanding of this process is crucial to optimize production yields. Nowadays, proteomics is one the most widely used “omic” approaches for studying stress conditions in biomining microorganisms [76]. In this study, proteomic analysis unveiled severe limitations in the central energetic metabolism of *A. thiooxidans* under extreme pH conditions, such as pH 0.7. These findings align with previous reports on *A. ferrooxidans*, which exhibited reduced metabolic activity at pH levels below 1.0, leading to compromised pH gradients and membrane potentials required for maintaining proton motive force [44]. Extreme acid stress has been identified as a significant threat to the survival of *Acidithiobacillus* strains [45,46,51].

Understanding the cellular processes occurring within *A. thiooxidans* under extremely acidic environments presents significant challenges, as many proteins, particularly those upregulated at pH 0.7, remain unidentified. To date, almost 50% of the *A. thiooxidans* genomes consist of hypothetical proteins, making it difficult to determine their mechanisms of action [77,78].

These findings partially circumvent the limited knowledge surrounding *A. thiooxidans*, as well as the critical necessity for conducting thorough and in-depth molecular-level investigations within the *Acidithiobacillus* genus. Such studies are essential to unravel the intricacies and complexities that exist within this genus in order to gain a deeper understanding of the genetic diversity, evolutionary relationships, and ecological roles of the *Acidithiobacillus* species, which may unlock eco-friendly industrial processes such as bioleaching, bioremediation, or biocatalysis.

## 4. Materials and Methods

### 4.1. Media and Strain Isolation

*A. thiooxidans* Lo.19II-12 was isolated from sludge from Rio Tinto (Huelva, Spain). An amount of 1 g of sludge samples was first enriched into 100 mL of DSMZ 71 (*Acidithiobacillus thiooxidans* medium) at 30 °C and 200 rpm for 7 days and grown onto *Thiobacillus* agar pH 4.0 supplemented with bromocresol green (BCG, 0.008 g/L) as pH indicator for 30 days at 30 °C [79]. *Thiobacillus* agar per liter consisted of the following components: (NH_4_)_2_SO_4_ (0.40 g), MgSO_4_·7 H_2_O (0.50 g), CaCl_2_ (0.25 g), KH_2_PO_4_ (4.00 g), FeSO_4_ (0.01 g), and Na_2_S_2_O_3_ (5.00 g).

### 4.2. A. thiooxidans DNA Isolation and Genome Sequencing

DNA isolation was carried out from a 200 mL liquid culture of *A. thiooxidans* Lo.19II-12 grown in modified DSMZ 35 medium (0.10 g/L NH_4_Cl, 3.00 g/L KH_2_PO_4_, 0.10 g/L MgCl_2_·6H_2_O, 0.14 g/L CaCl_2_·2H_2_O; pH 3.0) (https://www.dsmz.de/dsmz (accessed on 1 June 2023)) supplemented with 10% (*w*/*v*) powdered sulfur, at 30 °C and 200 rpm for 5 days, as described by Bergamo et al. [80]. Briefly, cells were centrifuged at 10,700× *g* for 1 min, washed twice with 5 mL of TE buffer (10 mM Tris-HCl, 1 mM EDTA; pH 8.0) and once with 1 mL of TAS buffer (50 mM Tris-HCl, 50 mM EDTA, 150 mM NaCl; pH 8.0). Cell pellet was resuspended into breaking mix buffer (50 µL 10% SDS, 150 µL 20 mg/mL Proteinase K, 300 µL TAS buffer), and incubated at 50 °C for 1 h, prior to being treated with 4 μL of RNase (10 mg/mL) for 1 h at 37 °C. A phenol-CIA (chloroform: isoamilic alcohol 24:1) extraction was carried out for protein elimination. For further molecular identification, *16S rRNA* gene was amplified using the 27F and 1492R primers [60] and sequenced at Instrumental Techniques Laboratory (University of León) following standard procedures (Table 2).

Extracted total DNA was sent to Macrogen (Humanizing Genomics Macrogen, Seoul, Republic of Korea) for Pacbio and Illumina sequencing, de novo assembly, and annotation. Functional analysis of *A. thiooxidans* NCBI genome was carried out using RAST server (Rapid Annotation using Subsystem Technology) (www.rast.theseed.org/FIG/rast.cgi (accessed on 5 March 2021)) [81,82].

### 4.3. 16S-5S Ribosomal Cluster Alignment and Analysis

The sequence of the *16S-5S rRNA* cluster of the strain Lo.19II-12 was compared to corresponding sequences of 103 *Acidithiobacillus* strains available from GenBank database. Multiple alignments of the corresponding sequences were performed by the CLUSTAL X algorithm [83] from the beginning of the *16S rRNA* gene (5′GAACNNNAGAGTTTGATCC3′) to the end of the *5S rRNA* gene (5′GAAAGTCGGTCACCGCCAGAC3′). Phylogenetic relationships were inferred by using the neighbor-joining method [40]. Evolutionary distances were computed using the maximum composite likelihood method [41]. Evolutionary analyses were conducted in MEGA7 v.7.0.26 [42] and the tree was visualized using iTOL v.6 [43].

### 4.4. A. thiooxidans Growth Determination by qPCR

An amount of 200 mL of modified DSMZ 35 medium was inoculated in triplicate with 1 mL of a 5-day pre-grown culture and incubated at 30 °C and 200 rpm for 11 days. Samples of 1.5 mL were taken daily and filtered through sterile filter paper. An amount of 1 mL of the filtered culture was centrifuged at 10,700× *g* for 10 min and pellets were washed with 100 µL of H_2_O and resuspended in 20 µL of sterile H_2_O. Samples were stored at 80 °C until use. At the end of the incubation, cell suspensions were quantified by qPCR all together.

The qPCR reactions were performed in an Mx3005P qPCR System (Stratagene) using SYBR TB Green Premix Ex Taq (Takara). qPCR reactions were carried out by triplicate in a 20 μL final volume containing 1× TB Green Premix Ex Taq (Takara), 200 nM of each primer (F_16S and R_16S, Table 2), and 5 µL of a 1:10 diluted culture. Cells were disrupted during the initial denaturation step. The qPCR cycling protocol was as follows: 1× initial denaturation step at 95 °C for 2 min; followed by 40× cycles of (i) 95 °C for 20 s; (ii) 20 s at 62 °C; and 1× a final extension of 1 min at 72 °C.

Melting curve assays were performed from 55 to 95 °C. Specific amplification was confirmed by a single peak in the melting curve. A standard curve of the Ct values vs. initial DNA concentration was calculated using total DNA with a concentration from 2.5 fg to 25 ng, and it was used to determine the concentration of the unknown samples, based on their Ct values [84]. Nuclease-free water was used as non-template control (NTC) and not inoculated DSMZ 35 medium was utilized as negative control. Next, the amount of DNA in the unknown sample (fg) is split into the number of copies of the 16S rRNA gene that exists in the initial sample (2 copies, according to genome sequences deposited in NCBI database), and is transformed by the length of the template (in bp) by the following formula:

$$copies=\frac{fg\;\left(DNA\right)\cdot \;N_{A}}{length\;\left(bp\right)\cdot \;M_{BP}}$$

where *N_A_* is Avogadro’s Number (6.022 × 10^23^ molecules/mol); *M_BP_* is the average weight of a base pair (bp), which is 660 g/mol; *fg* (*DNA*) is the amount of DNA based on its Ct; and length is the length of the template (182 bp) [44].

### 4.5. Analysis of pH Stress Conditions in A. thiooxidans by Reverse Transcription PCR (RT-PCR)

Differential expression of four well-known stress genes was checked to analyze pH stress conditions in *A. thiooxidans*: *rpoH*, *groEL2*, *dnaJ*, and *dnaK*; and *sdoA* expression was used as control [45,46,47]. By triplicate, 200 mL of modified DSMZ 35 medium supplemented with 1% (*w*/*v*) sulfur were inoculated with 1 mL of a 7-day pre-grown culture, and incubated at 30 °C and 200 rpm for 5 days. Cultures were then collected by centrifugation at 9600× *g* for 10 min and pellets were resuspended in 50 mL of DSMZ 35 at pH 3.0. Each sample was spread out into 5 different tubes containing 10 mL each. Samples were centrifuged at 9600× *g* for 10 min, resuspended in 50 mL of DSMZ 35 at the defined pH (0.5, 1.5, 3.0, 5.5, or 6.0), and incubated at 30 °C and 200 rpm for 30 min or 60 min. Samples were collected by centrifugation, treated with 1 mL of TRIzol reagent (Invitrogen), and incubated at RT for 5 min prior to the addition of 0.3 mL of chloroform. Samples were centrifuged at 17,000× *g* and 4 °C for 15 min. The upper phase was transferred to a new tube, treated with 0.55 volumes of isopropanol, and incubated at RT for 15 min. Samples were centrifuged at 17,000× *g* and 4 °C for 10 min. The pellets were washed with ethanol 70% and resuspended into 50 µL of nuclease-free water. Once the RNA was extracted, 1 µg was treated with 1 μL of DNAse I, RNase free (Thermo Scientific, Waltham, MA, USA) prior to the incubation at 37 °C for 30 min. Finally, 500 ng of the treated RNA was used as a template for 5× PrimeScript RT Master Mix (Takara, San Jose, CA, USA), and the reaction was incubated at 37 °C for 15 min. RT-PCR reactions were carried out in a final volume of 20 µL, containing 1× TB Green Premix Ex Taq (Takara) and 100 nM forward and reverse primers (Table 2). Cycling parameters are listed in Table 3.

Melting peaks were visualized to check the specificity of the amplification. The results obtained for each gene were normalized to the expression of *sdoA* gene. Three biologicals, with three technical replicates, were used. Relative gene expression was determined with the formula fold induction, 2^−ΔΔCt^, where:

$$\Delta \Delta Ct=\Delta Ct\;\left(EC\right)-\Delta Ct\;\left(CC\right)$$

$$\Delta Ct=Ct_{TG}-Ct_{RG}$$

*Ct* (cycle threshold) value is based on the threshold crossing point of individual fluorescence traces of each sample. *CC* is the control condition (pH 3.0) and *EC* are the experimental conditions. On the other hand, *TG* are the target genes (*groEL2*, *dnaJ*, *dnaK*, and *rpoH*) and *RG* is the reference gene (*sdoA*). The genes analyzed were considered significantly up- or down-regulated when differences of expression ratio were higher than 2 or lower than 0.5, respectively.

### 4.6. A. thiooxidans Proteome Analysis under pH Stress by 2D DIGE Analysis

Cultures were developed into 12 L Pyrex bottles containing 10 L of DSMZ35 liquid media supplemented with 0.1% sulfur. Cultures were developed at RT under constant agitation using a standard bar magnet and stirrer. Bottles were closed using a rubber stopper with two perforations through which silicone tubes were passed. Sterile air (80 L/h) was introduced through one of the tubes, previously passed through a 0.45-micron filter, using an aquarium pump and a plastic diffuser to prevent acid corrosion. The air left the bottle through the second silicone tube. Cultures were inoculated at 0.5% (*v*/*v*) with a 5-day grown pre-culture. The culture was grown for 65 h (middle of the exponential phase of growth) at RT, divided into 15 samples (650 mL per sample), centrifuged at 10,700× *g* for 10 min, and resuspended in 50 mL of DSMZ 35 medium at pH 0.7, 3.0, or 6.0, prior to the incubation at 30 °C and 200 rpm for 90 min. Protein extraction was carried out by following Barreiro et al. [85] protocol. Briefly, pellets were resuspended into 800 µL of 50 mM Tris-HCl pH 7.2 supplemented with a protease inhibitor mix (COMPLETE, Roche, Basel, Switzerland), and transferred to a FastProtein BLUE tube (BIO 101). Cell disruption was carried out in a Savant Bio 101 FastPrep FP120 cell disruption system (MP Biomedicals, Santa Ana, CA, USA) for 3 time intervals of 30 s at a speed ratio of 6.5 m/s, and 1 min resting on ice. Samples were centrifuged at 16,000× *g* for 10 min to remove cell debris and silica matrix prior to the incubation with Benzonase (Merck, Darmstadt, Germany) at 37 °C for 30 min. Proteins were concentrated by precipitation with 9 volumes of acetone and incubated at −20 °C O/N. Samples were then centrifuged at 14,000× *g* and 4 °C for 15 min. Protein pellets were air dried and resuspended in 100 µL of rehydration buffer (8 M urea, 2% [*w*/*v*] CHAPS [3-[(3-cholamidopropyl)dimethylammonio]-1-propanesulfonate], 0.01% bromophenol blue). Protein concentrations of the protein extracts were determined by the Bradford method [86]. DIGE analysis was performed based on the description of Vasco-Cárdenas et al. (2013). Briefly, 2D-Clean Kit™ (GE Healthcare, Chicago, IL, USA) was used to remove the disturbing components from protein samples, prior to resuspending them in resuspension buffer (8 M urea and 4% *w*/*v* CHAPS). Protein labeling was carried out by using a Cy dye ratio of 400 pmol/μL of dye per 50 μg of protein (50 µg Cy3, 50 µg Cy5, 25 µg Cy2, and 350 µg crude protein extract) for 30 min on ice in the dark. Labeling reaction was stopped by adding 1 µL of 10 mM lysine to each sample and incubating them on ice in the dark for 10 min. Samples were combined according to the experimental design, and up to 350 μL of rehydration buffer supplemented with 1% IPG buffer (GE Healthcare) and 80 mM dithithreitol (DTT) were added for the isoelectrofocusing (IEF) process. For IEF procedure, precast IPG strips with linear pH gradients of 3.0 to 10.0 were used in an IPGphor isoelectric focusing unit (GE Healthcare). Proteins were focused at 20 °C according to the following program: 1 h, 0 V; 12 h, 30 V; 2 h, 60 V; 1 h, 500 V; 30 min gradient until 8000 V, and 8000 V until 50,000 kVh. Focused IPG gels were equilibrated twice for 15 min in a buffer containing 50 mM Tris-HCl (pH 8.8), 6 M urea, 30% (*v*/*v*) glycerol, 2% (*w*/*v*) sodium dodecyl sulfate, and 1% (*w*/*v*) DTT. For the second equilibration step, DTT was replaced by 4% (*w*/*v*) iodoacetamide. The second dimension was run in SDS-12.5% polyacrylamide gels in an Ettan Dalt apparatus (Amersham) as recommended by the manufacturer, and gels were subsequently scanned by Ettan Dige Imager Software 1.0 (GE Healthcare) and stained with Coomassie brilliant blue [87]. Difference protein expression was analyzed with (i) *p*-value < 0.05, (ii) difference of expression ratio > 2, and <−2, (iii) at least appeared in 70% of gel images, and (iv) with a minimal volume higher than 4000. False discovery rate (FDR) was applied.

Protein spots were excised from gels and digested with modified trypsin (Promega, Madison, WI, USA) as described by [88], and peptide mass fingerprints were determined with a MALDI-tof-tof (mod. 4800, Applied Biosystem, Waltham, MA, USA) and analyzed using the MASCOT server (https://www.matrixscience.com/search_form_select.html (accessed on 3 June 2021)) [89]. A 4700 Proteomics analyzer calibration mixture (Cal Mix 5, AB Sciex) was used as external calibration. All MS spectra were internally calibrated using peptides from the auto-digested trypsin. A custom database was developed by joining the Uniprot γ-proteobacteria database (date 7 March 2021), every *Acidithiobacillus* strain proteome sequence from NCBI (date 12 March 2021), and every possible ORF from the *A. thiooxidans* Lo.19II-12 genome obtained by RanSEPS software v.2.7. Search parameters for peptide mass fingerprints and tandem MS spectra obtained were as follows: (i) custom database was used (950,740 sequences, 311,457,478 residues); (ii) fixed and variable modifications were considered (Cys as S carbamidomethyl derivative, and oxidized methionine); (iii) one missed cleavage site was allowed; (iv) precursor tolerance was 150 ppm and MS/MS fragment tolerance was 0.3 Da; and (v) peptide charge 1+; (vi) trypsin as the enzyme [90].

## 5. Conclusions

This study has yielded significant contributions in two pivotal realms: the introduction of a novel technology for assessing the growth of *Acidithiobacillus* and an in-depth elucidation of *A. thiooxidans*’ response to exceedingly acidic conditions. Firstly, the introduction of this innovative technique for monitoring *Acidithiobacillus* growth equips researchers with a valuable investigative tool to scrutinize the behavior and adaptive responses of these microorganisms, both in industrial and laboratory settings. Secondly, the proteomic analysis has revealed crucial insights into the intricate molecular mechanisms governing Acidithiobacillus’ reaction to environments characterized by extreme acidity.

The differential expression of various proteins associated with central energetic metabolism, carbon fixation, energy metabolism, nitrogen fixation, and stress response underscores the profound influence of pH on these fundamental cellular processes. Similarly, the RT-PCR studies elucidate that *A. thiooxidans* Lo.19II-12 can activate stress response systems, such as chaperones, when exposed to moderately neutral pH (pH 6.0). However, no activation of these genes is observed under extremely acidic pH conditions (pH 0.5), which may also be indicative of a potential cellular collapse. These findings not only enrich our understanding of *Acidithiobacillus*’ adaptive strategies but also provide a robust foundation for further research aimed at optimizing biomining systems and enhancing environmental bioremediation practices.

Nowadays, the industrial application of *Acidithiobacillus* members lacks comprehensive control over bacterial growth, cellular viability, or the intricate molecular mechanisms governing the processes. Implementing rigorous growth control measures, along with an exhaustive comprehension of the species and processes involved in heap bioleaching, holds the potential to significantly enhance process yields, rendering these systems more economically and industrially viable.

## Figures and Tables

**Table 1:** Relative transcript levels of the selected genes were determined by RT-PCR using *sdo*A as reference gene. Samples were recovered at 30 and 60 min of incubation at pH 0.5, 1.5, 3.0 (control condition, bold underlined font), 5.5, and 6.0. Data shown represent the average (with SD) from three independent experiments (biological replicates). The analyzed genes were considered significantly up- (light grey) or down-regulated (dark grey) when changes in their expression were higher than 2 or lower than 0.5, respectively.

Time	pH	2^−∆∆Ct^			
		*groEL2*	*dnaJ*	*rpoH*	*dnaK*
**30 min**	0.5	**0.00 ± 0.01**	**0.31 ± 0.52**	No Ct	No Ct
	1.5	**0.00 ± 0.00**	**0.00 ± 0.00**	**2.09 ± 4.46**	No Ct
	**3.0**	1.00 ± 0.00	1.00 ± 0.00	1.00 ± 0.00	1.00 ± 0.00
	5.5	0.50 ± 0.063	**0.41 ± 0.02**	**0.18 ± 0.00**	**0.37 ± 0.01**
	6.0	0.52 ± 0.009	0.78 ± 0.05	**0.48 ± 0.09**	0.60 ± 0.08
**60 min**	0.5	No Ct	No Ct	No Ct	No Ct
	1.5	**0.00 ± 0.05**	0.71 ± 0.51	0.94 ± 1.00	No Ct
	**3.0**	1.00 ± 0.00	1.00 ± 0.00	1.00 ± 0.00	1.00 ± 0.00
	5.5	1.59 ± 0.45	1.67 ± 1.09	1.26 ± 1.14	**0.23 ± 0.01**
	6.0	0.92 ± 0.43	**2.34 ± 1.34**	**4.70 ± 2.93**	0.54 ± 0.06

**Table 2:** Oligonucleotides used in PCR, qPCR, and RT-PCR assays in this work.

Gene	Primer Name	Primer Sequence	Amplicon Size (bp)	Reference
*16S rRNA*	27F	5′AGAGTTTGATCCTGGCTCAG3′	1500	Peace et al. 1994 [60]
	1492R	5′GGTTACCTTGTTACGACTT3′		
*16S rRNA*	F_16S	5′AGAGTTTGATCCTGGCT3′	182	This work
	R_16S	5′CGATTCTTTACCGAGTGG3′		
*dnaJ*	F_*dnaJ*	5′TCGAAGTCAAAGTGCCGGCCGGAGTAGATA3′	80	This work
	R_*dnaJ*	5′GCCGCGTTCCCCAGCTCCCCTTCACCATTCAGAC3′		
*dnaK*	F_*dnaK*	5′GGAAGGCGACAAGGTCAAGG3′	262	This work
	R_*dnaK*	5′CGCGCACTTCCACCCAGG3′		
*groEL*	F_*groEL2*	5′ACTGCGGCTATCTGTCGTCCTA3′	141	This work
	R_*groEL2*	5′GATCTGGCCGCCTCCTCTA3′		
*rpoH*	F_*rpoH*	5′TGCGCAACTGGCGTATTGTCAAAGTGGCTACCA3′	126	This work
	R_*rpoH*	5′CTTCAGCAATGGCCGCACTTTCCTCACCACTCAAC3′		
*sdoA*	F_*sdoA*	5′CGAGGAAAATCTGCAGGTTGAGTGG3′	154	This work
	R_*sdoA*	5′CGTTATAGATCTGGGCGAAAGTT3′		

**Table 3:** Cycling parameters of the RT-PCR for the five selected genes.

Cycles	*groEL2*	*dnaJ*	*rpoH*	*dnaK*	*sdoA*
**×1**	95 °C; 2 min				
**×40**	95 °C; 20 s				
	60 °C; 20 s	62 °C; 20 s	62 °C; 20 s	60 °C; 20 s	58 °C; 20 s
**×1**	72 °C; 1 min				

## Data Availability

Not applicable.

## Supplementary Materials

The supporting information can be downloaded at: https://www.mdpi.com/article/10.3390/ijms241713391/s1.
