# Supplementary material for: Molecular Identification and Acid Stress Response of an Acidithiobacillus thiooxidans Strain Isolated from Rio Tinto (Spain)
Source: Int J Mol Sci. 2023 Aug 29;24(17):13391. doi: 10.3390/ijms241713391 (PMC10487802; doi:10.3390/ijms241713391)
Supplement: Supplementary file 1 [file ijms-24-13391-s001.zip › ijms-2552981-supplementary.pdf]

Table S1. 16S rRNA sequences of the SOB isolates and BLASTn-based identification based on the percentage of identity.

Isolate	Sequence	Identification	Per. Identity
**De.02II-12**	CGCAGCACACCCCCAAAACCCCCCGTATGGCTCAGATTGAACGCTAGCGGCATGCTTTACACATGCAAGTCGAACGGCAGCGCGGGTAACCTGGCGGCGAGTGGCGAAC<br>GGGTGAGTAATACATCGGAACGTGCCCTGTGATGGGGGATAACTACGCGAAAGCGTAGCTAATACCGCATACGACCTACGGGTGAAAGTGGGGGATCGCAAGACCTCACG<br>TCATAGGAGCGGCCGATGGCGGATTAGCTAGTTGGTGAGGTAAAGGCTTACCAAGGCAACGATCCGTAGCTGGTCTGAGAGGACGACCAGCCACACTGGGACTGAGACAC<br>GGCCCAGACTCCTACGGGAGGCAGCAGTGGGGAATCTTGGACAATGGGGGCAACCCTGATCCAGCAATGCCGCGTGTGGGATGAAGGCCTTCGGGTTGTAAACCACTTTT<br>GGCGGGGGCGAAATATCGAGTGCTAATACCATTCGGTGATGACGGTACCCGCAGAATAAGCACCGGCTAACTACGTGCCAGCAGCCGCGGTAATACGTAGGGTGCAAGCG<br>TTAATCGGAATTACTGGGCGTAAAGCGTGCGCAGGCGGTTAGGTAAGACAGATGTGAAATTCCCGGGCTCAACCTGGGAACTGCATTTGTGACTGCCTGACTAGAGTGCG<br>GCAGAGGGGAGTGGAATTCCGCGTGTAGCAGTGAAATGCGTAGATATGCGGAGGAACACCGATGGCGAAGGCAACTCCCTGGGCCTGCACTGACGCTCATGCACGAAAGC<br>GTGGGGAGCAAACAGGATTAGATACCCTGGTAGTCCACGCCCTAAACGATGTCGACTAGTTGTTGGACGGGTTACTGTTCAGTAACGAAGCTAACGCGTGAAGTCGACCG<br>CCTGGGGAGTACGGCCGCAAGGTTAAAACTCAAAGGAATTGACGGGGACCCGCACAAGCGGTGGATGATGTGGTTTAATTCGATGCAACGCGAAAAACCTTACCTACCCT<br>TGACATGCCAGGAACTTACCAGAGATGGTTTGGTGCTCGAAAGAGAGCCTGGACACAGGTGCTGCATGGCTGTCGTCAGCTCGTGTCGTGAGATGTTGGGTTAAGTCCCG<br>CAAC	*Thiomonas* sp.	100%
**De.02II-34**	GTGCGGGACTTAACCCAACATCTCACGACACGAGCTGACGACAGCCATGCAGCACCTGTGTCCAGGCTCTCTTTCGAGCACCAAACCATCTCTGGTAAGTTCCTGGCATG<br>TCAAGGGTAGGTAAGGTTTTTCGCGTTGCATCGAATTAAACCACATCATCCACCGCTTGTGCGGGTCCCCGTCAATTCCTTTGAGTTTTAACCTTGCGGCCGTACTCCCC<br>AGGCGGTCGACTTCACGCGTTAGCTTCGTTACTGAACAGTAACCCGTCCAACAACTAGTCGACATCGTTTAGGGCGTGGACTACCAGGGTATCTAATCCTGTTTGCTCCC<br>CACGCTTTCGTGCATGAGCGTCAGTGCAGGCCCAGGGAGTTGCCTTCGCCATCGGTGTTCCTCCGCATATCTACGCATTTCACTGCTACACGCGGAATTCCACTCCCCTC<br>TGCCGCACTCTAGTCAGGCAGTCACAAATGCAGTTCCCAGGTTGAGCCCGGGAATTTCACATCTGTCTTACCTAACCGCCTGCGCACGCTTTACGCCCAGTAATTCCGAT<br>TAACGCTTGCACCCTACGTATTACCGCGGCTGCTGGCACGTAGTTAGCCGGTGCTTATTCTGCGGGTACCGTCATCACCGAATGGTATTAGCACTCGATATTTCGCCCCC<br>GCCAAAAGTGGTTTACAACCCGAAGGCCTTCATCCCACACGCGGCATTGCTGGATCAGGGTTGCCCCCATTGTCCAAGATTCCCCACTGCTGCCTCCCGTAGGAGTCTGG<br>GCCGTGTCTCAGTCCCAGTGTGGCTGGTCGTCCTCTCAGACCAGCTACGGATCGTTGCCTTGGTAAGCCTTTACCTCACCAACTAGCTAATCCGCCATCGGCCGCTCCTA<br>TGACGTGAGGTCTTGCGATCCCCCACTTTCACCCGTAGGTCGTATGCGGTATTAGCTACGCTTTCGCGTAGTTATCCCCCATCACAGGGCACGTTCCGATGTATTACTCA<br>CCCGTTCGCCACTCGCCGCCAGGTTACCCCGCGCTGCCGTTCGACTTGCATGTGTAAAGCATGCCGC	*Thiomonas* sp.	99.90%
**Lo.19II-1**	TGCCTAACACATGCAAGTCGAACGGTAACAGGTCTTCGGATGCTGACGAGTGGCGGACGGGTGAGTAATGCGTAGGAATCTGTCTTTGAGTGGGGGACAACCCAGGGAAA<br>CTTGGGCTAATACCGCATAAGCCCTGAGGGGGAAAGCGGGGGATCTTCGGACCTCGCGCTGGAAGAGGAGCCTACGTCTGATTAGCTAGTTGGTAGGGTAAAGGCCTACC<br>AAGGCGACGATCGGTAGCTGGTCTGAGAGGACGACCAGCCACACTGGGACTGAGACACGGCCCAGACTCCTACGGGAGGCAGCAGTGGGGAATTTTTCGCAATGGGGGCA<br>ACCCTGACGAAGCAATGCCGCGTGAATGAAGAAGGCCTTCGGGTTGTAAAGTTCTTTCGTGGAGGACGAAAAGGTGGGTGCTAATAACGCCTGCTGTTGACGTGAATCCA<br>AGAAGAAGCACCGGCTAACTCCGTGCCAGCAGCCGCGGTAATACGGGGGGTGCAAGCGTTAATCGGAATCACTGGGCGTAAAGGGTGCGTAGGCGGTGCATTAGGTCTGT<br>CGTGAAATCCCCGGGCTCAACCTGGGAATGGCGGTGGAAACCGGTGTACTAGAGTATGGGAGAGGGTGGTGGAATTCCAGGTGTAGCGGTGAAATGCGTAGAGATCTGGA<br>GGAACATCAGTGGCGAAGGCGGCCACCTGGCCCAATACTGACGCTGAGGCACGAAAGCGTGGGGAGCAAACAGGATTAGATACCCTTGGTAGTCCACGCCCTAAACGATG<br>AATACTAGATGTTTGGTGCCCAAGCGTACTGAGTGTCGTAGCTAACGCGATAAGTATTCCGCCTGGGAAGTACGGCCGCAAGGTTAAAACTCAAAGGAATTGACGGGGGC<br>CCGCACAAGCGGTGGAGCATGTGGTTTAATTCGATGCAACGCGAAGAACCTTACCTGGGCTTGACATGTCTGGAATCCTGCAGAGATGCGGGAGTGCCCTTCGGGGAATC<br>AGAACACAGGTGCTGCATGGCTGTCGTCAGCTCGTGTC	*Acidithiobacillus* sp.	99.81%

**Lo.19II-2**	GCTCAGATTGAACGCTGGCGGCATGCCTAACACATGCAAGTCGAACGGTAACAGGTCTTCGGATGCTGACGAGTGGCGGACGGGTGAGTAATGCGTAGGAATCTGTCTTT<br>TAGTGGGGGACAACCCAGGGAAACTTGGGCTAATACCGCATGAGCCCTGAGGGGGAAAGCGGGGGATCTTCGGACCTCGCGCTAAGGGAGGAGCCTACGTCTGATTAGCT<br>AGTTGGTAGGGTAAAGGCCTACCAAGGCGACGATCAGTAGCTGGTCTGAGAGGACGACCAGCCACACTGGGACTGAGACACGGCCCAGACTCCTACGGGAGGCAGCAGTG<br>GGGAATTTTTCGCAATGGGGGCAACCCTGACGAAGCAATGCCGCGTGGATGAAGAAGGCCTTCGGGTTGTAAAGTCCTTTCGTGGAGGACGAAAAGGCGGGTTCTAATAC<br>AATCTGCTGTTGACGTGAATCCAAGAAGAAGCACCGGCTAACTCCGTGCCAGCAGCCGCGGTAATACGGGGGGTGCAAGCGTTAATCGGAATCACTGGGCGTAAAGGGTG<br>CGTAGGCGGTACGTTAGGTCTGTCGTGAAATCCCCGGGCTCAACCTGGGAATGGCGGTGGAAACCGGCGGACTAGAGTATGGGAGAGGGTGGTGGAATTCCAGGTGTAGC<br>GGTGAAATGCGTAGAGATCTGGAGGAACATCAGTGGCGAAGGCGGCCACCTGGCCCAATACTGACGCTGAGGCACGAAAGCGTGGGGAGCAAACAGGATTAGATACCCTG<br>GTAGTCCACGCCCTAAACGATGAATACTAGATGTTTGGTGCCACGCGTACTGAGTGTCGTAGCTAACGCGATAAGTATTCCGCCTGGGAAGTACGGCCGCAAGGTTAAAA<br>CTCAAAGGAATTGACGGGGGCCCGCACAAGCGGTGGAGCATGTGGTTTAATTCGATGCAACGCGAAGAACCTTACCTGGGCTTGACATGTCCGGAATTCTGCAGAGATGC<br>GGGAGTGCCCTTCGGGGAATCGGAACACAGGTGCTGCATGGCTGTCGTCAGCTCGTGTCGTGAGATGTTGGGTTAAGTCCCGCA	*Acidithiobacillus* sp.	100%
**Lo.19II-12**	TGCCTAACACATGCAAGTCGAACGGTAACAGGTCTTCGGATGCTGACGAGTGGCGGACGGGTGAGTAATGCGTAGGAATCTGTCTTTGAGTGGGGGACAACCCAGGGAAA<br>CTTGGGCTAATACCGCATAAGCCCTGAGGGGGAAAGCGGGGGATCTTCGGACCTCGCGCTGGAAGAGGAGCCTACGTCTGATTAGCTAGTTGGTAGGGTAAAGGCCTACC<br>AAGGCGACGATCGGTAGCTGGTCTGAGAGGACGACCAGCCACACTGGGACTGAGACACGGCCCAGACTCCTACGGGAGGCAGCAGTGGGGAATTTTTCGCAATGGGGGC<br>AACCCTGACGAAGCAATGCCGCGTGAATGAAGAAGGCCTTCGGGTTGTAAAGTTCTTTCGTGGAGGACGAAAAGGTGGGTGCTAATAACGCCTGCTGTTGACGTGAATCC<br>AAGAAGAAGCACCGGCTAACTCCGTGCCAGCAGCCGCGGTAATACGGGGGGTGCAAGCGTTAATCGGAATCACTGGGCGTAAAGGGTGCGTAGGCGGTGCATTAGGTCTG<br>TCGTGAAATCCCCGGGCTCAACCTGGGAATGGCGGTGGAAACCGGTGTACTAGAGTATGGGAGAGGGTGGTGGAATTCCAGGTGTAGCGGTGAAATGCGTAGAGATCTGG<br>AGGAACATCAGTGGCGAAGGCGGCCACCTGGCCCAATACTGACGCTGAGGCACGAAAGCGTGGGGAGCAAACAGGATTAGATACCCTGGTAGTCCACGCCCTAAACGATG<br>AATACTAGATGTTTGGTGCCAAGCGTACTGAGTGTCGTAGCTAACGCGATAAGTATTCCGCCTGGGAAGTACGGCCGCAAGGTTAAAACTCAAAGGAATTGACGGGGGCC<br>CGCACAAGCGGTGGAGCATGTGGTTTAATTCGATGCAACGCGAAGAACCTTACCTGGGCTTGACATGTCTGGAATCCTGCAGAGATGCGGGAGTGCCCTTCGGGGAATCA<br>GAACACAGGTGCTGCATGGCTGTCGTCAGCTCGTGT	*Acidithiobacillus* sp.	99.90%
**Lo.15III-1**	ATGCCTAACACATGCAAGTCGAACGGTAACAGGTCTTCGGATGCTGACGAGTGGCGGACGGGTGAGTAATGCGTAGGAATCTGTCTTTGAGTGGGGGACAACCCAGGGAA<br>ACTTGGGCTAATACCGCATAAGCCCTGAGGGGGAAAGCGGGGGATCTTCGGACCTCGCGCTGGAAGAGGAGCCTACGTCTGATTAGCTAGTTGGTAGGGTAAAGGCCTAC<br>CAAGGCGACGATCGGTAGCTGGTCTGAGAGGACGACCAGCCACACTGGGACTGAGACACGGCCCAGACTCCTACGGGAGGCAGCAGTGGGGAATTTTTCGCAATGGGGGC<br>AACCCTGACGAAGCAATGCCGCGTGAATGAAGAAGGCCTTCGGGTTGTAAAGTTCTTTCGTGGAGGACGAAAAGGTGGGTGCTAATAACGCCTGCTGTTGACGTGAATCC<br>AAGAAGAAGCACCGGCTAACTCCGTGCCAGCAGCCGCGGTAATACGGGGGGTGCAAGCGTTAATCGGAATCACTGGGCGTAAAGGGTGCGTAGGCGGTGCATTAGGTCTG<br>TCGTGAAATCCCCGGGCTCAACCTGGGAATGGCGGTGGAAACCGGTGTACTAGAGTATGGGAGAGGGTGGTGGAATTCCAGGTGTAGCGGTGAAATGCGTAGAGATCTGG<br>AGGAACATCAGTGGCGAAGGCGGCCACCTGGCCCAATACTGACGCTGAGGCACGAAAGCGTGGGGAGCAAACAGGATTAGATACCCTGGTAGTCCACGCCCTAAACGATG<br>AATACTAGATGTTTGGTGCCAAGCGTACTGAGTGTCGTAGCTAACGCGATAAGTATTCCGCCTGGGAAGTACGGCCGCAAGGTTAAAACTCAAAGGAATTGACGGGGGCC<br>CGCACAAGCGGTGGAGCATGTGGTTTAATTCGATGCAACGCGAAGAACCTTACCTGGGCTTGACATGTCTGGAATCCTGCAGAGATGCGGGAGTGCCCTTCGGGGAATCA<br>GAACACAGGTGCTGCATGGCTGTCGTCCAGCTCGTGTCGT	*Acidithiobacillus* sp.	99.90%

**Lo.15III-2**	GCTAACACATGCAAGTCGAACGGTAACAGGTCTTCGGATGCTGACGAGTGGCGGACGGGTGAGTAATGCGTAGGAATCTGTCTTTGAGTGGGGGACAACCCAGGGAAACT<br>TGGGCTAATACCGCATAAGCCCTGAGGGGGAAAGCGGGGGATCTTCGGACCTCGCGCTGGAAGAGGAGCCTACGTCTGATTAGCTAGTTGGTAGGGTAAAGGCCTACCAA<br>GGCGACGATCGGTAGCTGGTCTGAGAGGACGACCAGCCACACTGGGACTGAGACACGGCCCAGACTCCTACGGGAGGCAGCAGTGGGGAATTTTTCGCAATGGGGGCAAC<br>CCTGACGAAGCAATGCCGCGTGAATGAAGAAGGCCTTCGGGTTGTAAAGTTCTTTCGTGGAGGACGAAAAGGTGGGTGCTAATATCGCCTGCTGTTGACGTGAATCCAAG<br>AAGAAGCACCGGCTAACTCCGTGCCAGCAGCCGCGGTAATACGGGGGGTGCAAGCGTTAATCGGAATCACTGGGCGTAAAGGGTGCGTAGGCGGTGCATTAGGTCTGTCG<br>TGAAATCCCCGGGCTCAACCTGGGAATGGCGGTGGAAACCGGTGTACTAGAGTATGGGAGAGGGTGGTGGAATTCCAGGTGTAGCGGTGAAATGCGTAGAGATCTGGAGG<br>AACATCAGTGGCGAAGGCGGCCACCTGGCCCAATACTGACGCTGAGGCACGAAAGCGTGGGGAGCAAACAGGATTAGATACCCTGGTAGTCCACGCCCTAAACGATGAAT<br>ACTAGATGTTTGGTGCCAAGCGTACTGAGTGTCGTAGCTAACGCGATAAGTATTCCGCCTGGGAAGTACGGCCGCAAGGTTAAAACTCAAAGGAATTGACGGGGGCCCGC<br>ACAAGCGGTGGAGCATGTGGTTTAATTCGATGCAACGCGAAGAACCTTACCTGGGCTTGACATGTCTGGAATCCTGCAGAGATGCGGGAGTGCCCTTCGGGGAATCAGAA<br>CACAGGTGCTGCATGGCTGTCGTCAGCTCG	*Acidithiobacillus* sp.	100%
**Lo.15III-14**	TGCCTAACACATGCAAGTCGAACGGTAACAGGTCTTCGGATGCTGACGAGTGGCGGACGGGTGAGTAATGCGTAGGAATCTGTCTTTGAGTGGGGGACAACCCAGGGAAA<br>CTTGGGCTAATACCGCATAAGCCCTGAGGGGGAAAGCGGGGGATCTTCGGACCTCGCGCTGGAAGAGGAGCCTACGTCTGATTAGCTAGTTGGTAGGGTAAAGGCCTACC<br>AAGGCGACGATCGGTAGCTGGTCTGAGAGGACGACCAGCCACACTGGGACTGAGACACGGCCCCAGACTCCTACGGGAGGCAGCAGTGGGGAATTTTTCGCAATGGGGGC<br>AACCCTGACGAAGCAATGCCGCGTGAATGAAGAAGGCCTTCGGGTTGTAAAGTTCTTTCGTGGAGGACGAAAAGGTGGGTGCTAATATCGCCTGCTGTTGACGTGAATCC<br>AAGAAGAAGCACCGGCTAACTCCGTGCCAGCAGCCGCGGTAATACGGGGGGTGCAAGCGTTAATCGGAATCACTGGGCGTAAAGGGTGCGTAGGCGGTGCATTAGGTCTG<br>TCGTGAAATCCCCGGGCTCAACCTGGGAATGGCGGTGGAAACCGGTGTACTAGAGTATGGGAGAGGGTGGTGGAATTCCAGGTGTAGCGGTGAAATGCGTAGAGATCTGG<br>AGGAACATCAGTGGCGAAGGCGGCCACCTGGCCCAATACTGACGCTGAGGCACGAAAGCGTGGGGAGCAAACAGGATTAGATACCCTGGTAGTCCACGCCCTAAACGATG<br>AATACTAGATGTTTGGTGCCAAGCGTACTGAGTGTCGTAGCTAACGCGATAAGTATTCCGCCTGGGAAGTACGGCCGCAAGGTTAAAACTCAAAGGAATTGACGGGGGCC<br>CGCACAAGCGGTGGAGCATGTGGTTTAATTCGATGCAACGCGAAGAACCTTACCTGGGCTTGACATGTCTGGAATCCTGCAGAGATGCGGGAGTGCCCTTCGGGGAATCA<br>GAACACAGGTGCTGCATGGCTGTCGTCAGCTCGTT	*Acidithiobacillus* sp.	100%

Table S2. List of cytoplasmic proteins picked and digested with trypsin in the A. thiooxidans proteome analysis under extreme acid and neutral pH conditions. Spot proteins are numbered on the figures 33 and 34. Experimental isoelectric point (pI) and molecular weight (Mw); the up- or down-regulation ratios in pH 0.7 vs 3.0, and pH 0.7 vs 6.0 conditions; the COG and KEGG numbers; and the functional classification by COG system are indicated. Those proteins up-regulated at pH 0.7 are highlighted in pink color.

pI	Mw (kDa)	0,7/3,0 Av. Ratio	0,7/6,0 Av. Ratio	Accesion No.	Description	COG No.	KEGG No.	EggNOG Category	Function
4.42	15.3	-5.14	-8.80	Lo19II-12_1440	50S ribosomal protein L7/L12	COG0222	K02935	J	Translation
4.43	40.0	-16.01	-16.52	Lo19II-12_1500	hypothetical protein	COG2067	K06076	I	Lipid transport and metabolism
4.63	54.5	-38.37	-40.79	Lo19II-12_2871	Enolase	COG0148	K01689	G	Central C metabolism
5.05	42.0	-5.12	-4.66		hypothetical protein				
5.06	10.0	-2.40	-3.39	Lo19II-12_3122	Major Carboxysome Shell Protein 1C	COG4577	K04027	E	CO2 fixation
5.06	41.2	-9.53	-11.35	Lo19II-12_873	Putative membrane transport protein	COG1494	K06076	G	Central C metabolism
5.13	43.8	-3.39	-2.71	Lo19II-12_569	hypothetical protein	-	-	R	
5.14	41.9	-3.57	-2.89		hypothetical protein				
5.19	71.1	-44.84	-35.13	Lo19II-12_8	GroEL chaperone	COG0459	K04077	O	Chaperone
5.21	57.6	-35.22	-33.67	Lo19II-12_3130	Ribulose bisphosphate carboxylase large chain	COG1850	K01601	G	CO2 fixation
5.22	22.	-19.76	-20.23	Lo19II-12_469	Putative inorganic diphosphatase	COG0221	K01507	C	Central C metabolism
5.23	50.73	-4.79	-5.68	Lo19II-12_987	Beta sliding clamp	COG0592	K03629	L	DNA repair
5.23	53.5	-6.94	-6.62	Lo19II-12_1596	Metalloprotease TldD	COG0312	K03568	S	
5.25	5.0	-4.79	-5.02	Lo19II-12_3129	Ribulose bisphosphate carboxylase small chain	COG4451	K01602	C	CO2 fixation
5.36	56.6	-33.55	-36.18	Lo19II-12_240	Glutamate synthase [NADPH] small chain	COG0493	K00266	E	N fixation
5.36	46.9	-5.77	-4.67	Lo19II-12_453	S-adenosylmethionine synthase	COG0192	K00789	H	Transport and metabolism
5.39	22.6	-8.44	-12.35	Lo19II-12_2388	ATP-dependent Clp protease	COG0740	K01358	O	Chaperone
5.41	42.5	3.04	3.31	Lo19II-12_569	**hypothetical protein**	-	-	R	
5.43	43.7	1.98	2.11	Lo19II-12_3295	**hypothetical protein**	COG2067	-	I	Lipid transport and metabolism
5.47	39.7	-6.58	-5.08	Lo19II-12_455	Adenosine kinase	COG0524	K00856	G	Central C metabolism
5.49	61.4	-26.52	-38.25	Lo19II-12_1033	ATP synthase subunit alpha	COG0056	K02111	C	Central C metabolism
5.53	28.5	-2.29	-2.25	Lo19II-12_2153	Putative metal-dependent hydrolase YcfH	COG0084	K03424	L	DNA repair
5.57	53.6	-24.57	-16.46	Lo19II-12_953	Glycerol kinase	COG0554	K00864	C	Central C metabolism
5.64	5.0	2.96	1.26	Lo19II-12_1161	**hypothetical protein**	COG3439	-	S	
5.68	24.6	-5.77	-4.86	Lo19II-12_879	Ribulose-phosphate 3-epimerase	COG0036	K01783	G	Central C metabolism
5.76	42.5	-2.27	-1.74		hypothetical protein				
5.89	44.4	-2.05	-2.07	Lo19II-12_2952	NADP-dependent malic enzyme	COG0281	-	C	Central C metabolism
6.00	21.5	-2.01	-2.52	Lo19II-12_2919	Ribosome-recycling factor	COG0233	K02838	J	Translation
6.06	53.6	-13.55	-12.8	Lo19II-12_407	SoxB	COG0737	K17224	F	S metabolism
6.11	53.7	-8.16	-7.66	Lo19II-12_2860	Dihydrolipoyl dehydrogenase	COG1249	-	C	Central C metabolism
6.18	67.2	-2.58	-3.07	Lo19II-12_407	Mannosylglucosyl-3-phosphoglycerate phosphatase	COG0737	K17224	F	Central C metabolism
6.22	53.4	-22.01	-21.28	Lo19II-12_407	SoxB	COG0737	K17224	F	S metabolism
6.28	22.9	-52.40	-48.63	Lo19II-12_2656	Superoxide dismutase [Fe]|	COG0605	K04564	P	Transport and metabolism
6.42	39.3	-28.15	-26.52	Lo19II-12_875	Glyceraldehyde-3-phosphate dehydrogenase	COG0057	K00134	G	Central C metabolism
6.84	5.0	5.33	2.60	Lo19II-12_431	**hypothetical protein**	-	-	S	

7.20	5.0	2.87	2.00	Lo19II-12_431	**hypothetical protein**	-	-	S	
7.44	28.4	13.16	9.65	Lo19II-12_462	**Parvulin-type peptidyl-prolyl cis-trans**	COG0760	K03769	O	Chaperone
9.89	15.4	3.23	3.21	Lo19II-12_154	**hypothetical protein**	cl21600	-	R	
10.06	25.2	19.4	15.91	Lo19II-12_1433	**Peptidoglycan-binding protein ArfA**	COG2885	K03286	M	Ammonia secretion

Table S3. Acidithiobacillus genomes used in the study, accession identifiers and metadata of geographic location and reference sequence from NCBI. Data was recovered from NCBI database and/or collected in this study. When not reported specifically in the literature, latitude and longitude were calculated for the city, province or prefecture available. Data was recovered from genome submission reports, listed publications and/or collected in this study. All available Acidithiobacillus strains were classified in phylogenetic groups utilizing their 16S-5S rRNA cluster sequences and based on Moya-Beltrán et al. (2021) and L. Li et al. (2019).

Strain	Current classification	New classification	Geographic location	Latitude	Longitude	Ref.Seq. (NCBI)
Acidithiobacillus albertensis DSM 14366	*A. albertensis*	*A. thiooxidans* sp. *albertensis*	Canada: Alberta	56.130366	-106.346771	GCF_001931655.1
Acidithiobacillus albertensis No 26	*A. albertensis*	*A. thiooxidans* sp. *albertensis*	Norway: Roeros	62.572197	11.376421	GCF_018853595.1
Acidithiobacillus caldus 6	*A. caldus*	*Fervidacidithiobacillus cladus*	South Africa: Barberton	-25.731957	31.067435	GCF_018854195.1
Acidithiobacillus caldus ATCC 51756	*A. caldus*	*Fervidacidithiobacillus cladus*	United Kingdom: England, North Warwickshire	52.565820	-1.683508	GCF_000175575.2
Acidithiobacillus caldus BC13	*A. caldus*	*Fervidacidithiobacillus cladus*	United Kingdom: England, Warwick	52.267135	-1.467522	GCF_018853575.1
Acidithiobacillus caldus C-SH12	*A. caldus*	*Fervidacidithiobacillus cladus*	Australia: Brisbane	-27.451349	153.023374	GCF_018854145.1
Acidithiobacillus caldus CV18-1	*A. caldus*	*Fervidacidithiobacillus cladus*	Argentina: Neuquen	-37.499821	-70.559946	GCF_018853985.1
Acidithiobacillus caldus DSM 8584	*A. caldus*	*Fervidacidithiobacillus cladus*	United Kingdom: England, North Warwickshire	52.565820	-1.683508	GCF_018853615.1
Acidithiobacillus caldus DX	*A. caldus*	*Fervidacidithiobacillus cladus*	China: Jiangxi	27.291818	115.282396	GCF_001756675.1
Acidithiobacillus caldus F	*A. caldus*	*Fervidacidithiobacillus cladus*	South Africa: Vosburg	-30.593549	22.852902	GCF_018853755.1
Acidithiobacillus caldus KU	*A. caldus*	*Fervidacidithiobacillus cladus*	United Kingdom: England, North Warwickshire	52.565820	-1.683508	GCF_018853545.1
Acidithiobacillus caldus MELC5	*A. caldus*	*Fervidacidithiobacillus cladus*	Chile: Antofagasta	-23.650999	-70.398208	GCF_018853765.1
Acidithiobacillus caldus MNG	*A. caldus*	*Fervidacidithiobacillus cladus*	South Africa: Western Cape	-33.769046	18.384170	GCF_018854235.1
Acidithiobacillus caldus MTH-04	*A. caldus*	*Fervidacidithiobacillus cladus*	China: Tenchong area, Yunnan province	24.923108	98.537724	GCF_001650235.3
Acidithiobacillus caldus SM-1	*A. caldus*	*Fervidacidithiobacillus cladus*	China: Shenzhen	35.861660	104.195397	GCF_000221025.1
Acidithiobacillus caldus VAN18-3	*A. caldus*	*Fervidacidithiobacillus cladus*	Argentina:Copahue	-37.817827	-71.097782	GCF_018853695.1

Acidithiobacillus caldus ZBY	*A. caldus*	*Fervidacidithiobacillus cladus*	Zambia: Chambishi	-12.648502	28.048678	GCF_001756725.1
Acidithiobacillus caldus ZJ	*A. caldus*	*Fervidacidithiobacillus cladus*	China: Fujian	25.719660	118.035825	GCF_001756745.1
Acidithiobacillus ferrianus MG	*A. ferrianus*	*A. ferrianus*	Grece: Island of Milos	36.698298	24.395061	GCF_010378095.1
Acidithiobacillus ferridurans ATCC 33020	*A. ferridurans*	*A. ferridurans*	Japan: Okayama prefecture, Ningyo-toge	35.314454	133.930699	GCF_018854555.1
Acidithiobacillus ferridurans B5	*A. ferridurans*	*A. ferridurans*	France: Carnoules	43.286006	6.226177	GCA_018854545.1
Acidithiobacillus ferridurans DSM 583	*A. ferridurans*	*A. ferridurans*	USA: Pennsylvania	40.786407	-79.781590	GCF_018854535.1
Acidithiobacillus ferridurans DSM 9465	*A. ferridurans*	*A. ferridurans*	Australia: Ghan	-25.172106	133.181165	GCF_018854615.1
Acidithiobacillus ferridurans JCM 18981	*A. ferridurans*	*A. ferridurans*	Japan:Okayama	35.316584	133.936757	GCF_003966655.1
Acidithiobacillus ferridurans MEL3	*A. ferridurans*	*A. ferridurans*	Czech Republic: Zlate Hory	50.250072	17.390357	GCF_018854635.1
Acidithiobacillus ferridurans Riv11	*A. ferridurans*	*A. ferridurans*	Montserrat: Soufriere Hills	16.711178	-62.177296	GCF_018854695.1
Acidithiobacillus ferriphilus A4	*A. ferriphilus*	*A. ferriphilus*	Chile: Andacollo	-30.235697	-71.083176	GCF_018854785.1
Acidithiobacillus ferriphilus C52	*A. ferriphilus*	*A. ferriphilus*	Chile: Combarbala	-31.178952	-71.003924	GCF_018854755.1
Acidithiobacillus ferriphilus DSM 100412	*A. ferriphilus*	*A. ferriphilus*	Montserrat: Galway's Soufriere	16.683991	-62.166924	GCF_018854775.1
Acidithiobacillus ferriphilus GT2	*A. ferriphilus*	*A. ferriphilus*	USA: Colorado	39.550265	-105.782111	GCF_020844025.1
Acidithiobacillus ferriphilus Malay	*A. ferriphilus*	*A. ferriphilus*	Malaysia: Pahang	3.837907	103.299028	GCF_018853855.1
Acidithiobacillus ferriphilus R1	*A. ferriphilus*	*A. ferriphilus*	Romania: Ilba	47.716301	23.350883	GCF_018854825.1
Acidithiobacillus ferriphilus Riv13	*A. ferriphilus*	*A. ferriphilus*	Montserrat: White River	16.742498	-62.187366	GCF_018854815.1
Acidithiobacillus ferriphilus SCUT-1	*A. ferriphilus*	*A. ferriphilus*	China: Dabaoshan, Shaoguan	24.813956	113.601370	GCF_019400025.1
Acidithiobacillus ferriphilus ST2	*A. ferriphilus*	*A. ferriphilus*	Spain: Rio Tinto	37.385491	-6.661808	GCF_018854875.1
Acidithiobacillus ferrivorans ACH	*A. ferrivorans*	*A. ferrivorans*	Chile: Tarapaca	-20.551766	-69.665004	GCF_018854855.1
Acidithiobacillus ferrivorans CF27	*A. ferrivorans*	*A. ferrivorans*	USA: Idaho	44.068202	-114.742041	GCF_000750615.1
Acidithiobacillus ferrivorans DSM 22755	*A. ferrivorans*	*A. ferrivorans*	Norway:Kongsberg	59.672480	9.650242	GCF_018853935.1
Acidithiobacillus ferrivorans PQ33	*A. ferrivorans*	*A. ferrivorans*	Peru: Pasco	-10.396075	-75.448301	GCF_001857665.2
Acidithiobacillus ferrivorans PRJEB5721	*A. ferrivorans*	*A. ferrivorans*	USA: Idaho	44.068202	-114.742041	GCF_900174455.1

Acidithiobacillus ferrivorans SS3	*A. ferrivorans*	*A. ferrivorans*	Russia: Norilsk	69.355790	88.189294	GCF_000214095.2
Acidithiobacillus ferrivorans XJFY6S-08	*A. ferrivorans*	*A. ferrivorans*	China:xinjiang	40.408679	85.624802	GCF_016250455.1
Acidithiobacillus ferrivorans YL15	*A. ferrivorans*	*A. ferrivorans*	China: Tibet	31.800201	98.029925	GCF_001685225.1
Acidithiobacillus ferrooxidans ATCC 23270	*A. ferrooxidans*	*A. ferrooxidans*	USA: Pennsylvania	41.203322	-77.194525	GCF_000021485.1
Acidithiobacillus ferrooxidans ATCC 53993	*A. ferrooxidans*	*A. ferrooxidans*	Armenia: Alaverdi	41.102406	44.661526	GCF_000020825.1
Acidithiobacillus ferrooxidans BN	*A. ferrooxidans*	*A. ferrooxidans*	Zambia: Chambishi	-12.648334	28.047991	GCF_024606725.1
Acidithiobacillus ferrooxidans BY0502	*A. ferrooxidans*	*A. ferriphilus*	China: Gansu	37.505128	103.104235	GCF_001652185.1
Acidithiobacillus ferrooxidans BY-3	*A. ferrooxidans*	*A. ferrooxidans*	China: Baiyin, Gansu	37.505128	103.104235	GCF_010577825.1
Acidithiobacillus ferrooxidans BYM	*A. ferrooxidans*	*A. ferrooxidans*	China: Baiyin	36.550227	104.141456	GCF_019856615.1
Acidithiobacillus ferrooxidans CCM 4253	*A. ferrooxidans*	*A. ferrooxidans*	Czech Republic: Zlate Hory	50.264120	17.394476	GCF_003233765.1
Acidithiobacillus ferrooxidans CF3	*A. ferrooxidans*	*Acidithiobacillus ferruginosus*	USA: Idaho	44.001058	-114.279772	GCF_018854495.1
Acidithiobacillus ferrooxidans COP1	*A. ferrooxidans*	*A. ferrooxidans*	Wales			GCA_018854355.1
Acidithiobacillus ferrooxidans DLC-5	*A. ferrooxidans*	*A. ferrooxidans*	China: Heihe, Heilongjiang, Wudalianchi	48.748770	126.590359	GCA_000732185.1
Acidithiobacillus ferrooxidans DSM 16786	*A. ferrooxidans*	*A. ferrooxidans*	Chile			GCF_018853455.1
Acidithiobacillus ferrooxidans DX	*A. ferrooxidans*	*A. ferrooxidans*	China: Jiangxi	27.291818	115.282396	GCF_024606705.1
Acidithiobacillus ferrooxidans GD-0	*A. ferrooxidans*	*A. ferrooxidans*	China: Guangdong	24.815202	113.595877	GCF_024592575.1
Acidithiobacillus ferrooxidans GD-B	*A. ferrooxidans*	*A. ferrooxidans*	China: Guangdong	24.815202	113.595877	GCF_024606715.1
Acidithiobacillus ferrooxidans F221	*A. ferrooxidans*	*A. ferrooxidans*	Austria: Forstenau	47.379095	13.551586	GCF_018854265.1
Acidithiobacillus ferrooxidans Hel18	*A. ferrooxidans*	*A. ferrooxidans*	Germany: Helbra	51.554954	11.487649	GCF_001559335.1
Acidithiobacillus ferrooxidans NFP31	*A. ferrooxidans*	*A. ferrooxidans*	Japan: Tokyo, Miyake-jima	34.050178	139.310054	GCF_020887015.1
Acidithiobacillus ferrooxidans PQ505	*A. ferrooxidans*	*A. ferrooxidans*	Peru: Chaupimarca, Pasco	-10.396075	-75.448301	GCF_018854395.1

Acidithiobacillus ferrooxidans PQ506	*A. ferrooxidans*	*A. ferrooxidans*	Peru: Chaupimarca, Pasco	-10.396075	-75.448301	GCF_018854475.1
Acidithiobacillus ferrooxidans RVS1	*A. ferrooxidans*	*A. ferrooxidans*	Argentina: Neuquen	-37.499821	-70.559946	GCA_003931975.1
Acidithiobacillus ferrooxidans TFBk	*A. ferrooxidans*	*A. ferrooxidans*	Kazakhstan: Bakyrchik deposit	49.715132	81.588372	GCF_024626545.1
Acidithiobacillus ferrooxidans YNTRS-40	*A. ferrooxidans*	*A. ferrooxidans*	China: Yunnan	25.010194	98.509979	GCF_013462805.1
Acidithiobacillus ferrooxidans YQ_N3	*A. ferrooxidans*	*A. ferrooxidans*	China: Shandi River Basin, Yangquan city	37.861102	113.581121	GCF_020221655.1
Acidithiobacillus ferrooxidans YQH-1	*A. ferrooxidans*	*A. ferriphilus*	China: Heilongjiang	46.589310	125.103784	GCF_001418795.1
Acidithiobacillus sp. AMD consortium	*Acidithiobacillus sp.*	*A. ferridurans*	Canada: Sudbury	46.495470	-81.055800	GCF_008926505.1
Acidithiobacillus sp. ATCC 19703	*Acidithiobacillus sp.*	*A. thiooxidans*	Australia: Melbourne	-37.711044	144.972942	GCF_018853445.1
Acidithiobacillus sp. BN09_2	*Acidithiobacillus sp.*	*Igneacidithiobacillus copahuensis*	Argentina: Neuquen	-37.499821	-70.559946	GCF_018854135.1
Acidithiobacillus sp. CV18-1	*Acidithiobacillus sp.*	*Igneacidithiobacillus copahuensis*	Argentina: Neuquen	-37.499821	-70.559946	GCF_018854115.1
Acidithiobacillus sp. CV18-2	*Acidithiobacillus sp.*	*Igneacidithiobacillus copahuensis*	Argentina: Neuquen	-37.499821	-70.559946	GCF_018854095.1
Acidithiobacillus sp. CV18-4	*Acidithiobacillus sp.*	*Igneacidithiobacillus copahuensis*	Argentina: Neuquen	-37.499821	-70.559946	GCF_018854095.1
Acidithiobacillus sp. GG1_14	*Acidithiobacillus sp.*	*A. thiooxidans*	Montserrat: Saint Anthony	16.742498	-62.187366	GCF_018853465.1
Acidithiobacillus sp. GGI-221	*Acidithiobacillus sp.*		India	22.790786	79.703066	GCF_000179815.1
Acidithiobacillus sp. HP_11	*Acidithiobacillus sp.*	*A. thiooxidans*	USA: New York	43.125547	-78.370052	GCF_015100075.1
Acidithiobacillus sp. HP_2	*Acidithiobacillus sp.*	*A. thiooxidans*	USA: New York	43.125547	-78.370052	GCF_015100155.1
Acidithiobacillus sp. HP_6	*Acidithiobacillus sp.*	*A. thiooxidans*	USA: New York	43.125547	-78.370052	GCF_015100135.1
Acidithiobacillus sp. MC6.1	*Acidithiobacillus sp.*	*A. ferrivorans*	Antarctica: Marian Cove, King George Island	-62.214339	-58.746154	GCF_017165985.1
Acidithiobacillus sp. PG05	*Acidithiobacillus sp.*	*A. ferrooxidans*	Chile: Puerto Guadal, Aysen Region	-46.870139	-72.670224	GCF_017165965.1
Acidithiobacillus sp. RW2	*Acidithiobacillus sp.*	*A. thiooxidans*	United Kingdom: Wales-Trefriw	53.151257	-3.825580	GCF_018853815.1
Acidithiobacillus S30A2	*Acidithiobacillus sp.*					GCF_023277115.1
Acidithiobacillus sp. SH	*Acidithiobacillus sp.*	*A. thiooxidans*	Japan: Okayama, Seto-Inland Sea	34.290204	133.439979	GCF_002847505.1
Acidithiobacillus sp. VAN18_1	*Acidithiobacillus sp.*	*Igneacidithiobacillus copahuensis*	Argentina: Neuquen	-37.499821	-70.559946	GCF_018854015.1

Acidithiobacillus sp. VAN18_2	*Acidithiobacillus sp.*	*Igneacidithiobacillus copahuensis*	Argentina: Neuquen	-37.499821	-70.559946	GCF_018854025.1
Acidithiobacillus sulfuriphilus CJ-2	*A. sulfuriphilus*		Wales	53.055371	-3.858145	GCF_003721225.1
Acidithiobacillus thiooxidans A01	*A. thiooxidans*	*A. thiooxidans*	China: Jiangxi, Pingxiang	28.946464	117.578713	GCF_000559045.1
Acidithiobacillus thiooxidans A02	*A. thiooxidans*	*A. thiooxidans*	China: Jiangxi, Pingxiang	28.946464	117.578713	GCF_001705645.1
Acidithiobacillus thiooxidans ATCC 15494	*A. thiooxidans*	*A. thiooxidans*	USA			GCF_018854725.1
Acidithiobacillus thiooxidans ATCC 19377	*A. thiooxidans*	*A. thiooxidans*	England: Dorset	50.612788	-2.135306	GCF_009662475.1
Acidithiobacillus thiooxidans ATCC 21835	*A. thiooxidans*	*A. thiooxidans*	Japan: Gunma	36.390668	139.060406	GCF_018853915.1
Acidithiobacillus thiooxidans ATCC 8085	*A. thiooxidans*	*A. thiooxidans*	USA: New Jersey	40.056541	-74.308630	GCF_018854645.1
Acidithiobacillus thiooxidans BC51	*A. thiooxidans*	*A. thiooxidans*	United Kingdom:Warwickshire	52.269599	-1.547936	GCF_018853795.1
Acidithiobacillus thiooxidans BN09-1	*A. thiooxidans*	*A. thiooxidans*	Argentina: Neuquen	-37.499821	-70.559946	GCF_018854655.1
Acidithiobacillus thiooxidans BY-02	*A. thiooxidans*	*A. thiooxidans*	China: Gansu, Baiyin	36.545814	104.136650	GCF_001705725.1
Acidithiobacillus thiooxidans CLST	*A. thiooxidans*	*A. thiooxidans*	Chile: Salar de Gorbea, Atacama Region	-25.390183	-68.696151	GCF_002079865.1
Acidithiobacillus thiooxidans DMC	*A. thiooxidans*	*A. thiooxidans*	China: Hunan, Chenzhou	25.738462	113.028365	GCF_001705625.1
Acidithiobacillus thiooxidans DXS-W	*A. thiooxidans*	*A. thiooxidans*	China: Xinjiang	42.927298	93.673535	GCF_001705805.1
Acidithiobacillus thiooxidans GD1-3	*A. thiooxidans*	*A. thiooxidans*	China: Guangdong, Shaoguan	24.815202	113.595877	GCF_001705695.1
Acidithiobacillus thiooxidans JYC-17	*A. thiooxidans*	*A. thiooxidans*	China: Gansu, Baiyin	36.545814	104.136650	GCF_001705755.1
Acidithiobacillus thiooxidans VAN18-5	*A. thiooxidans*	*A. thiooxidans*	Argentina: Neuquen	-37.499821	-70.559946	GCF_018854715.1
Acidithiobacillus thiooxidans ZBY	*A. thiooxidans*	*A. thiooxidans*	Zambia: Chambishi	-12.648334	28.047991	GCF_001756595.1
Acidithiobacillus thiooxidans Lo.19II-12	*A. thiooxidans*	*A. thiooxidans*	Spain: Rio Tinto	37.690293	-6.561146	
